# Supplementary material for: Olfactory sensory neurons transiently express multiple olfactory receptors during development
Source: Mol Syst Biol. 2015 Dec 8;11(12):844. doi: 10.15252/msb.20156639 (PMC4704490; doi:10.15252/msb.20156639)
Supplement: Supplementary file 1 — Appendix [file MSB-11-844-s001.pdf]

Appendix

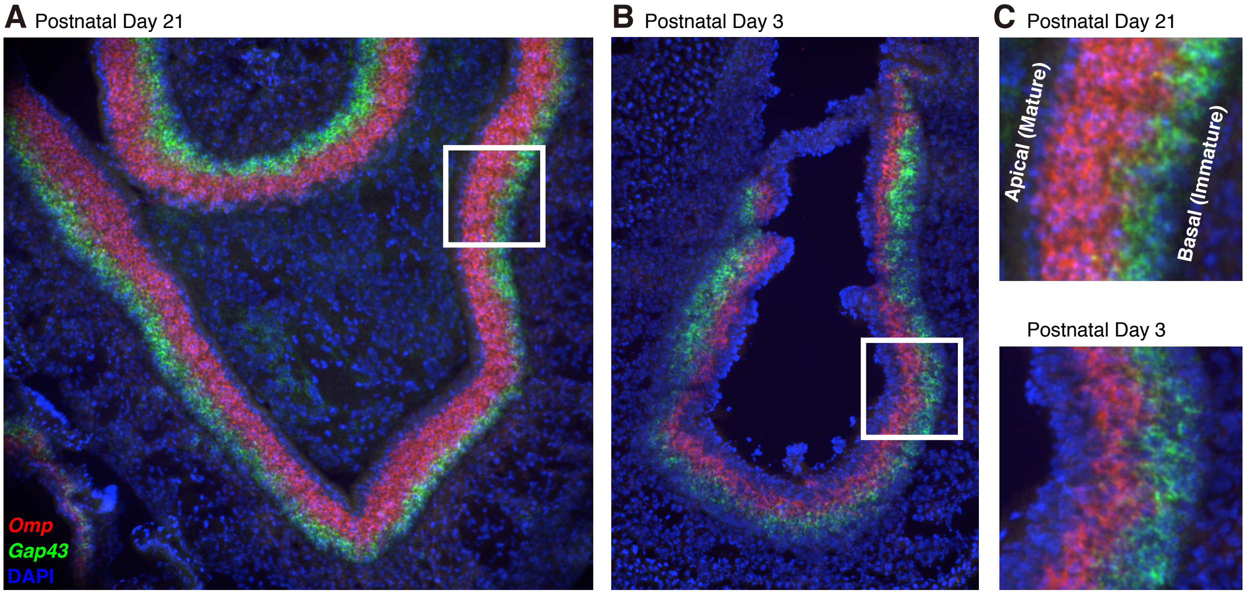

Appendix Figure S1. Two-color RNA *in situ* hybridization (green: *Gap43*, a marker for immature neurons; red: *Omp*, a marker for mature neurons; blue: DAPI) showed a higher immature-to-mature ratio in newborns. This is consistent with our sequencing data and published observations (Verhaagen et al. 1989). Panel (**C**) zooms in to areas in white boxes in (**A**) and (**B**).

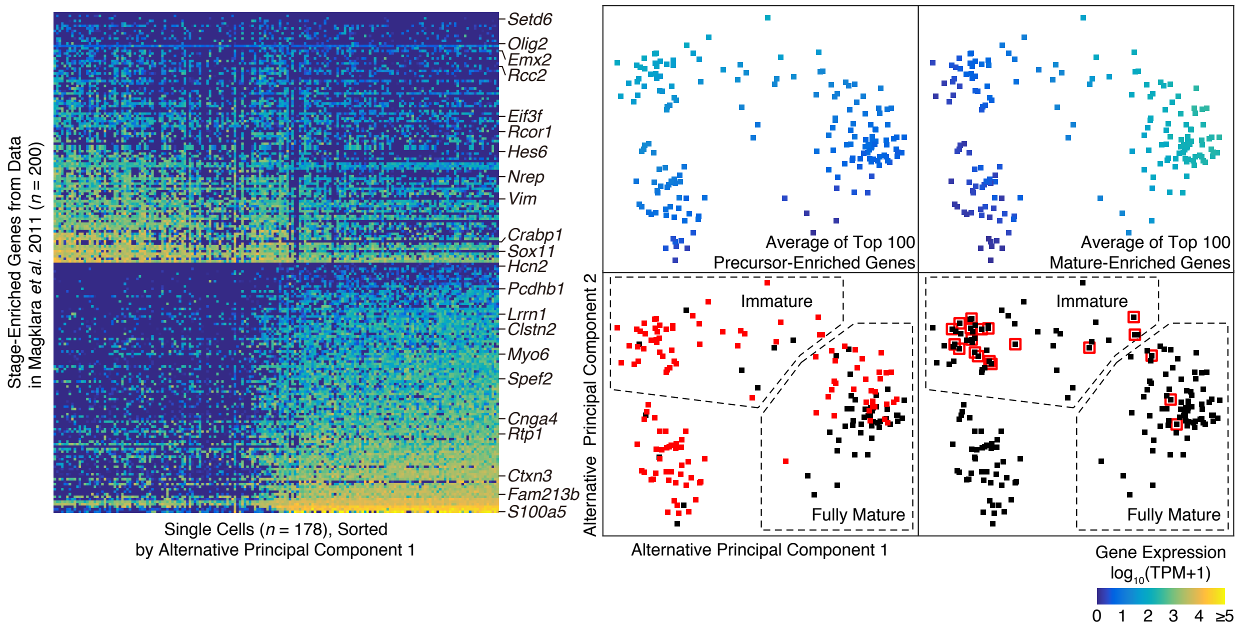

Appendix Figures S2. Our conclusions hold regardless of the choice of marker genes. We picked another set of genes (top 100 precursor-enriched genes and top 100 mature-enriched genes) from a recent study that conducted RNA sequencing on FACS-sorted samples (Magklara et al. 2011). Left: Genes were sorted first by categories (top: precursor-enriched, bottom: mature-enriched), and then by average expression across all cells. Right: Top two panels show the average profile for each of the two categories, calculated by taking the mean of the $\log_{10}$(TPM + 1) value for all genes in each category. Bottom left panel shows the age of the animals (red squares: cells from newborn mice; black squares: cells from adult mice). Bottom right panel shows multi-receptor neurons in red boxes.

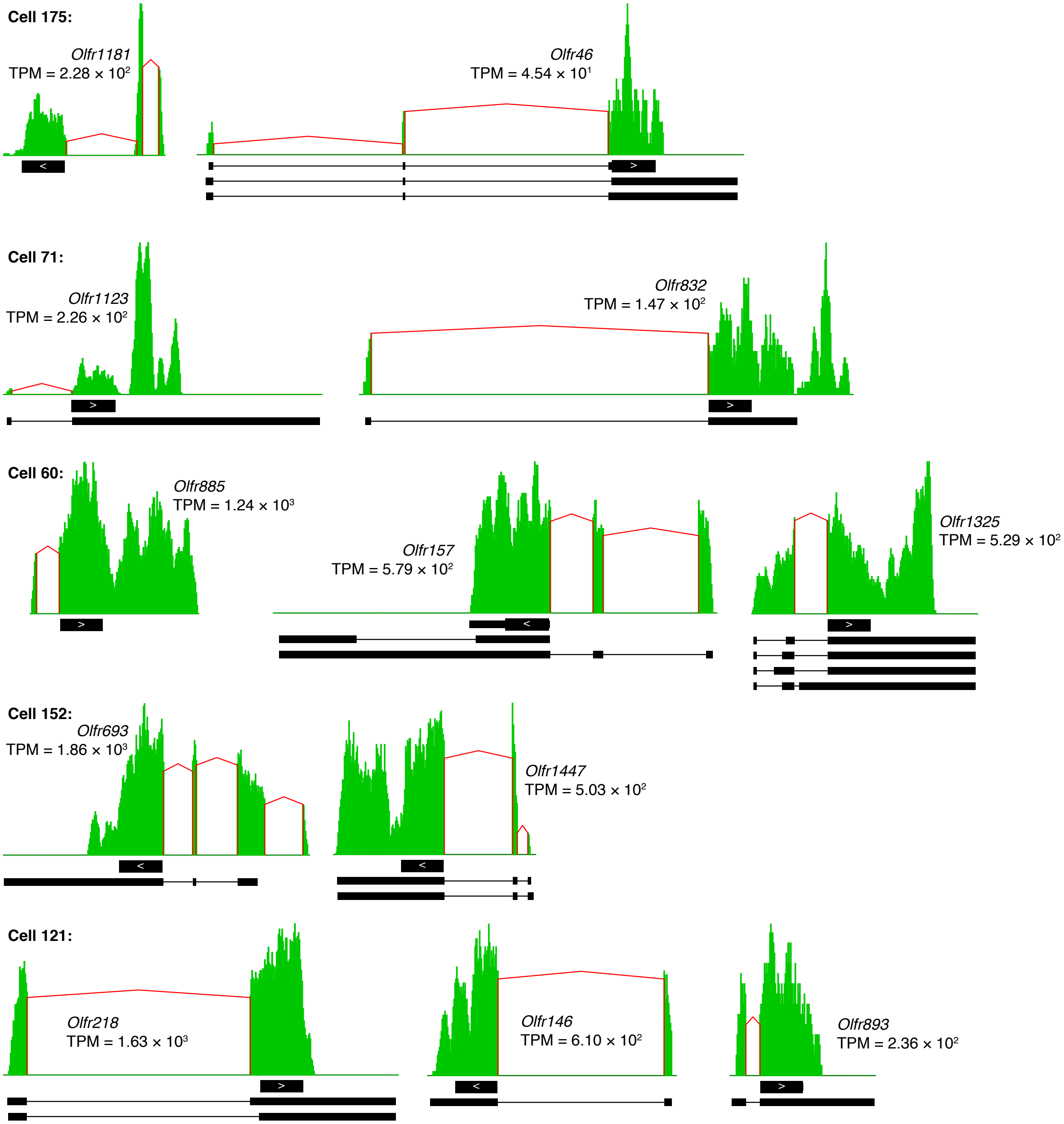

Appendix Figures S3. Coverage profiles of ORs in all 20 multi-receptor neurons: Part 1. Neurons are sorted by total OR expression as in Fig. 3D. Symbols have the same meaning as in Fig. 2C.

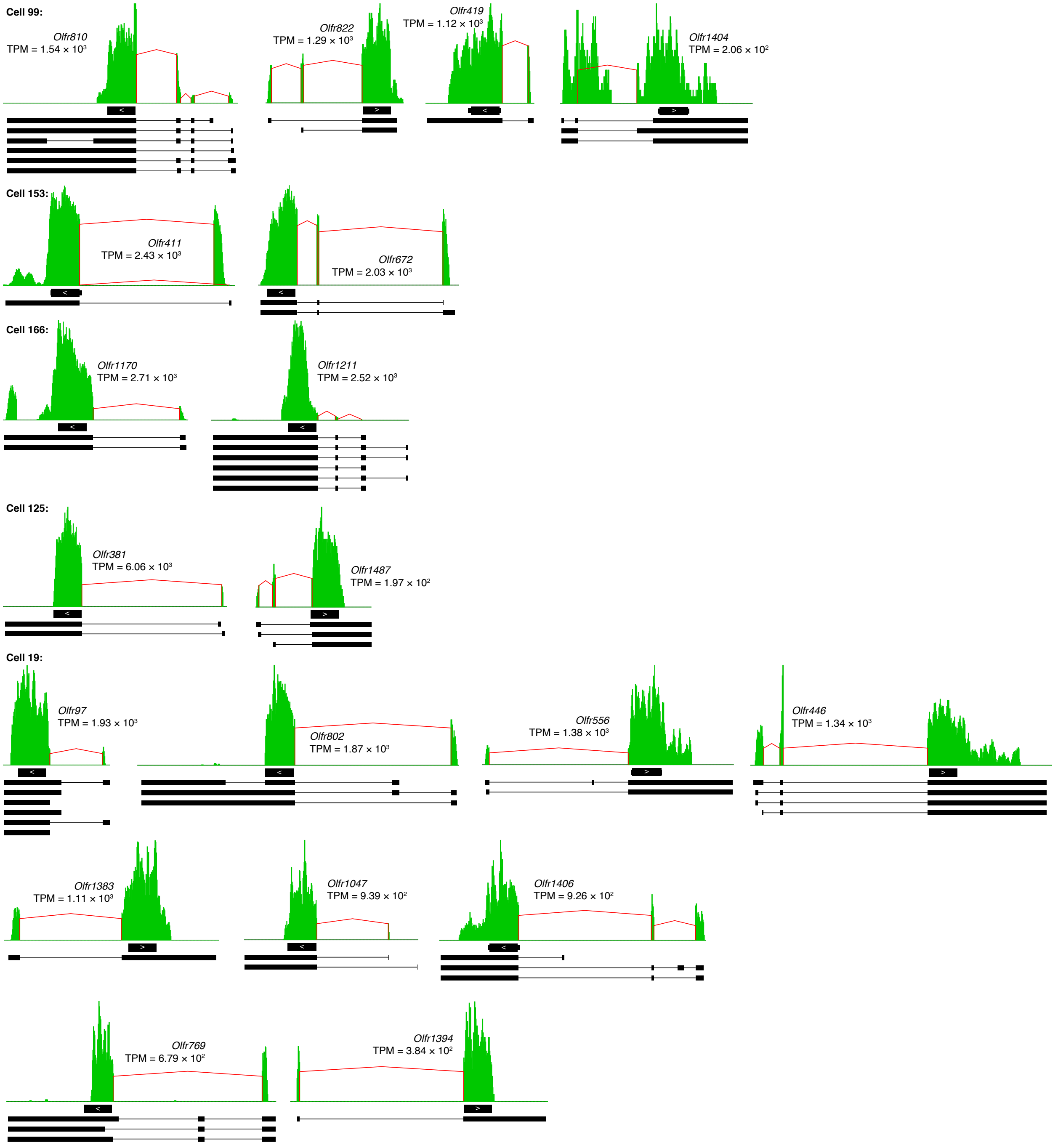

Appendix Figures S4. Coverage profiles of ORs in all 20 multi-receptor neurons: Part 2. Neurons are sorted by total OR expression as in Fig. 3D. Symbols have the same meaning as in Fig. 2C.

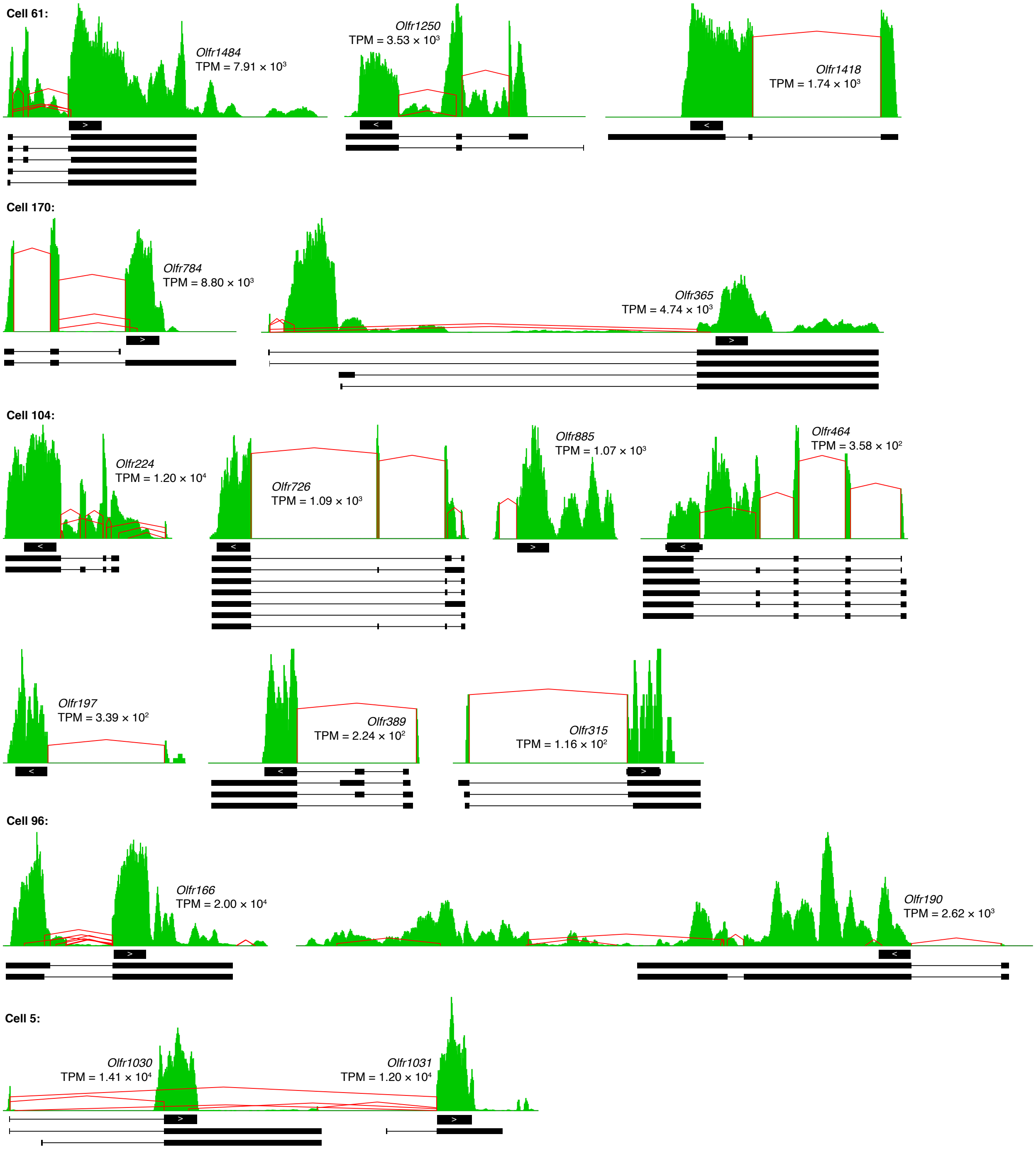

Appendix Figures S5. Coverage profiles of ORs in all 20 multi-receptor neurons: Part 3. Neurons are sorted by total OR expression as in Fig. 3D. Symbols have the same meaning as in Fig. 2C.

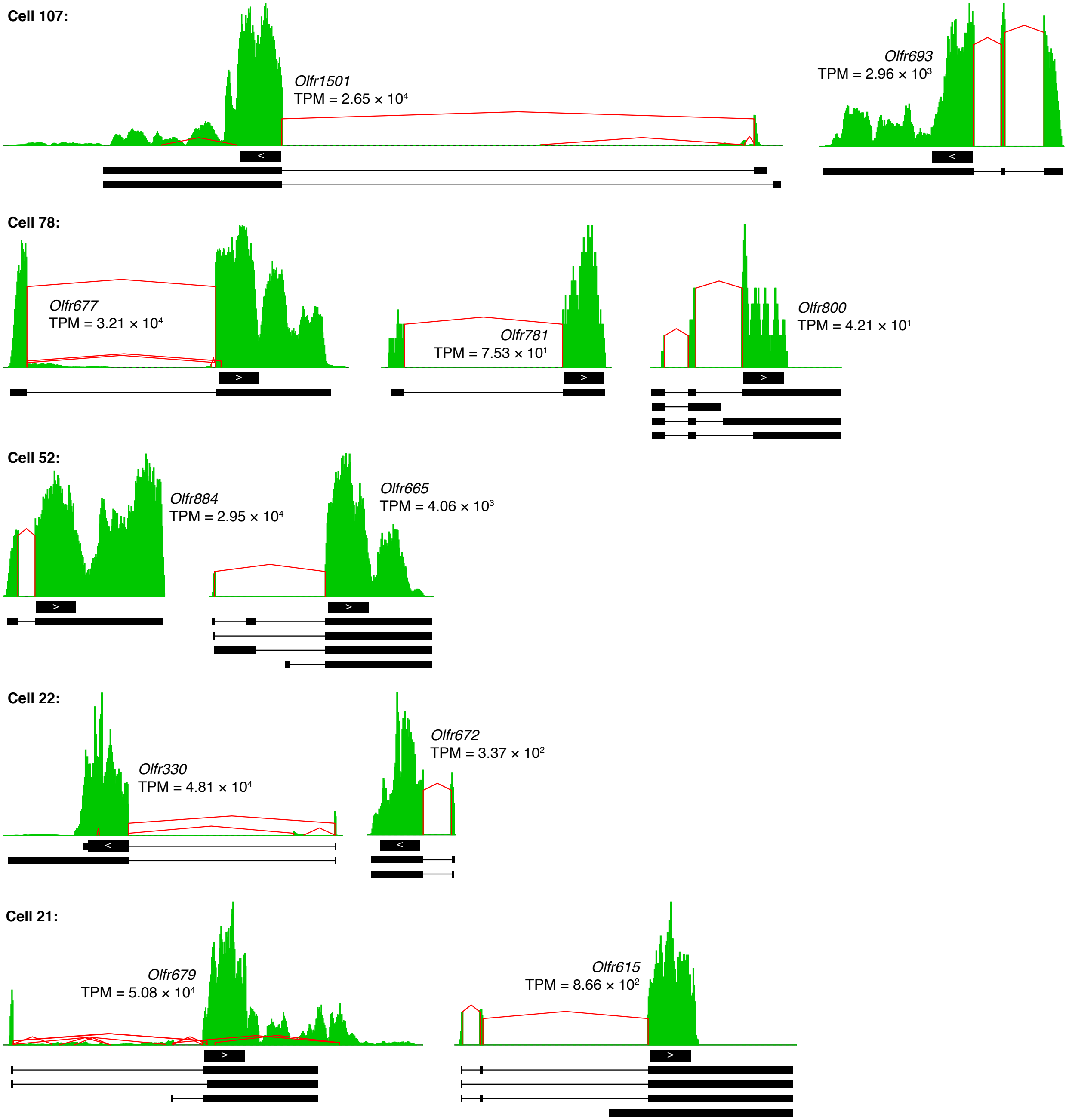

Appendix Figures S6. Coverage profiles of ORs in all 20 multi-receptor neurons: Part 4. Neurons are sorted by total OR expression as in Fig. 3D. Symbols have the same meaning as in Fig. 2C.

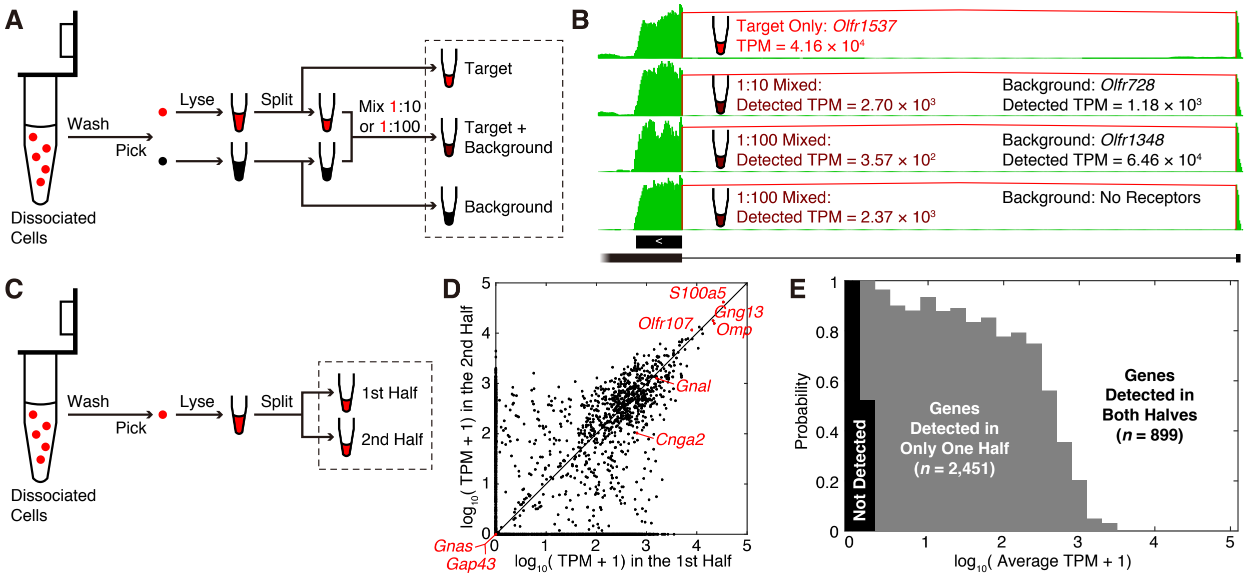

Appendix Figures S7. Control experiments examined the potential effects of contamination and of technical variability. (**A**) We conducted a control experiment in which a "target" cell, expressing a single OR *Olfr1537* at TPM = 4.16×10^4, was either reverse transcribed, amplified and sequenced alone, or processed as a 1:10 or 1:100 mixture with a "background" cell. (**B**) In all 3 mixtures, we detected the "target" OR *Olfr1537* regardless of the "background" cell. Symbols have the same meanings as in Fig. 2C. (**C**) To assess the extent of technical variations in single-cell transcriptomic sequencing, we conducted another control experiment in which a single cell was split into two halves and processed separately. (**D**) The two halves showed great consistency quantifying highly expressed genes (such as the OR *Olfr107*), but showed considerable noise detecting and quantifying lowly or intermediately expressed genes. Each dot denotes a single gene, with some example genes highlighted in red. The diagonal line denotes exact equality between the two halves. (**E**) Highly expressed genes were consistently detected by both halves (white), while lowly expressed genes experienced frequent "drop-outs" (gray, detection in only one half). The transition occurred at an expression level of TPM = 10^2 to 10^3 (corresponding to 0.01% to 0.1% of the transcriptome). Expression levels were calculated from the mean value of the two halves.

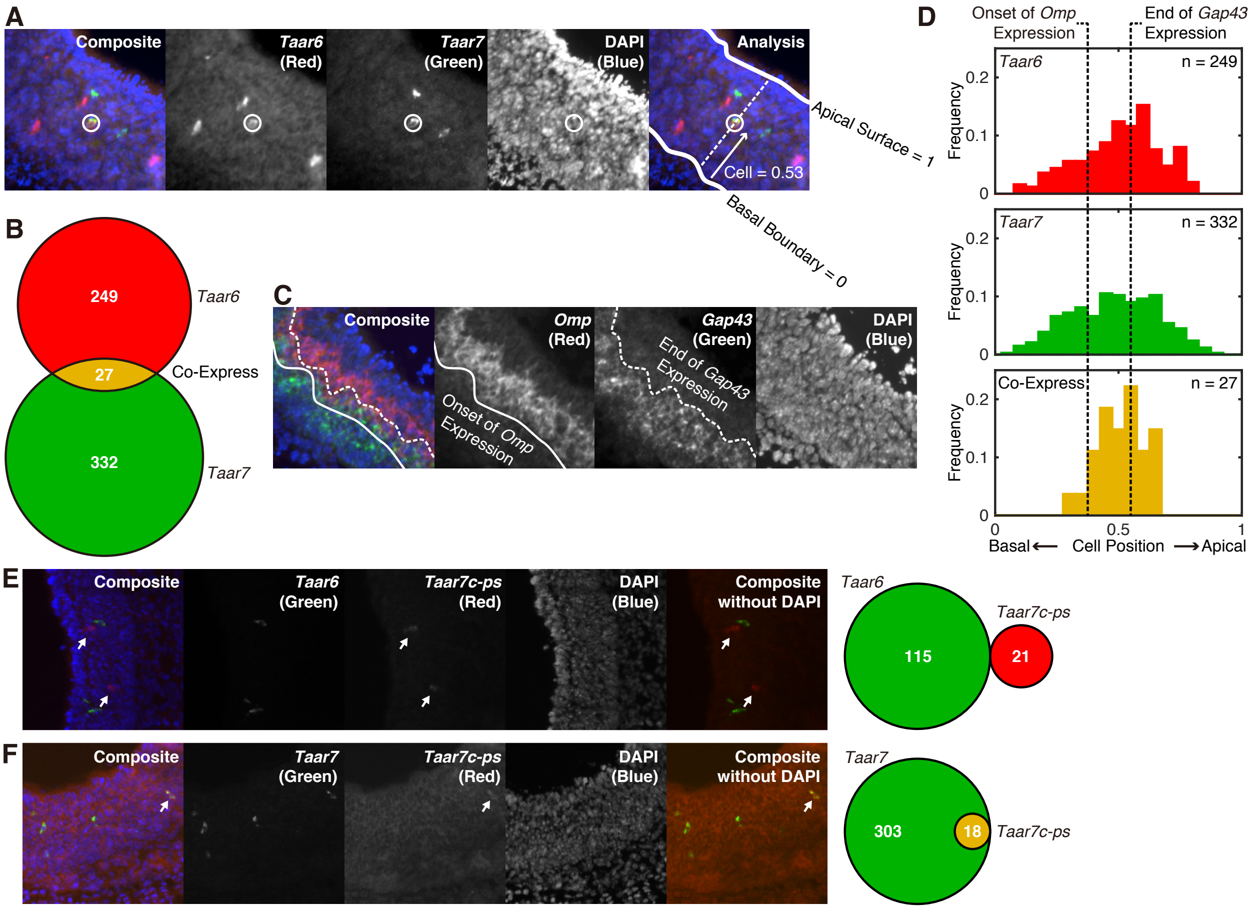

Appendix Figures S8. Co-expression of *Taar6* and *Taar7* in the olfactory epithelium of newborn mice. In tissue cryosections of newborn (postnatal day 3) mice, we found cells co-expressing two trace amine-associated receptors (TAARs); and they were concentrated at the transition between immature and mature stages, consistent with our deep-sequencing results on olfactory receptors. At this age, *Taar6* and the *Taar7* subfamily (*Taar7a/b/d/e/f*) are the most prevalent TAARs in the main olfactory epithelium (by counting 890 TAAR neurons in single-color RNA *in situ* hybridization, we estimated the following percentages: *Taar2*: 4%, *Taar3*: 3%, *Taar4*: 6%, *Taar5*: 4%, *Taar6*: 21%, *Taar7*: 51%, *Taar8*: 6%, *Taar9*: 6%). (**A**) We conducted two-color RNA *in situ* hybridization with published probes for *Taar6* (red) and for *Taar7* (green) (Liberles and Buck 2006). Nuclei were stained by DAPI (blue). In analysis, we quantified the relative position of each cell, with 0 denoting the basal boundary (more immature, thick white line) and 1 denoting the apical surface (more mature, thick white line). For example, the position of the circled cell (co-expressing *Taar6* and *Taar7*) is 0.53. (**B**) We found 27 cells that co-expressed *Taar6* and *Taar7*, which accounted for 10% of *Taar6*-expressing cells and 8% of *Taar7*-expressing cells. (**C**) To establish standards for inference of developmental stages, we performed two-color RNA *in situ* hybridization with probes for the mature marker *Omp* (red) and for the immature marker *Gap43* (green). At this stage, we found roughly equal number of cells expressing each marker and observed considerable overlap between the two. In a manner similar to (**A**), we quantified the onset of *Omp* expression to be at an average position of 0.38, and the end of *Gap43* expression was 0.55. (**D**) Histograms for the relative positions of cells expressing only *Taar6* (red), only *Taar7* (green), or both (yellow). Cells that express both are more

concentrated in the middle region, where neurons transition between the immature and mature stages. (**E**) We performed two-color RNA *in situ* hybridization with a *Taar6* probe (green) and a specific probe for the only pseudogene *Taar7c-ps* (red) in the *Taar7* family. No cells co-expressed *Taar6* and *Taar7c-ps*. (**F**) Two-color RNA *in situ* hybridization with a *Taar7* probe (green) and a specific probe for *Taar7c-ps* (red) revealed 100% co-localization of *Taar7c-ps with Taar7.* This can be explained by either the recognition of *Taar7c-ps* mRNA by the *Taar7* probe or the co-expression of *Taar7c-ps* with other *Taar7* members. Arrows in (**E**) and (**F**) point to *Taar7c-ps*+ cells.

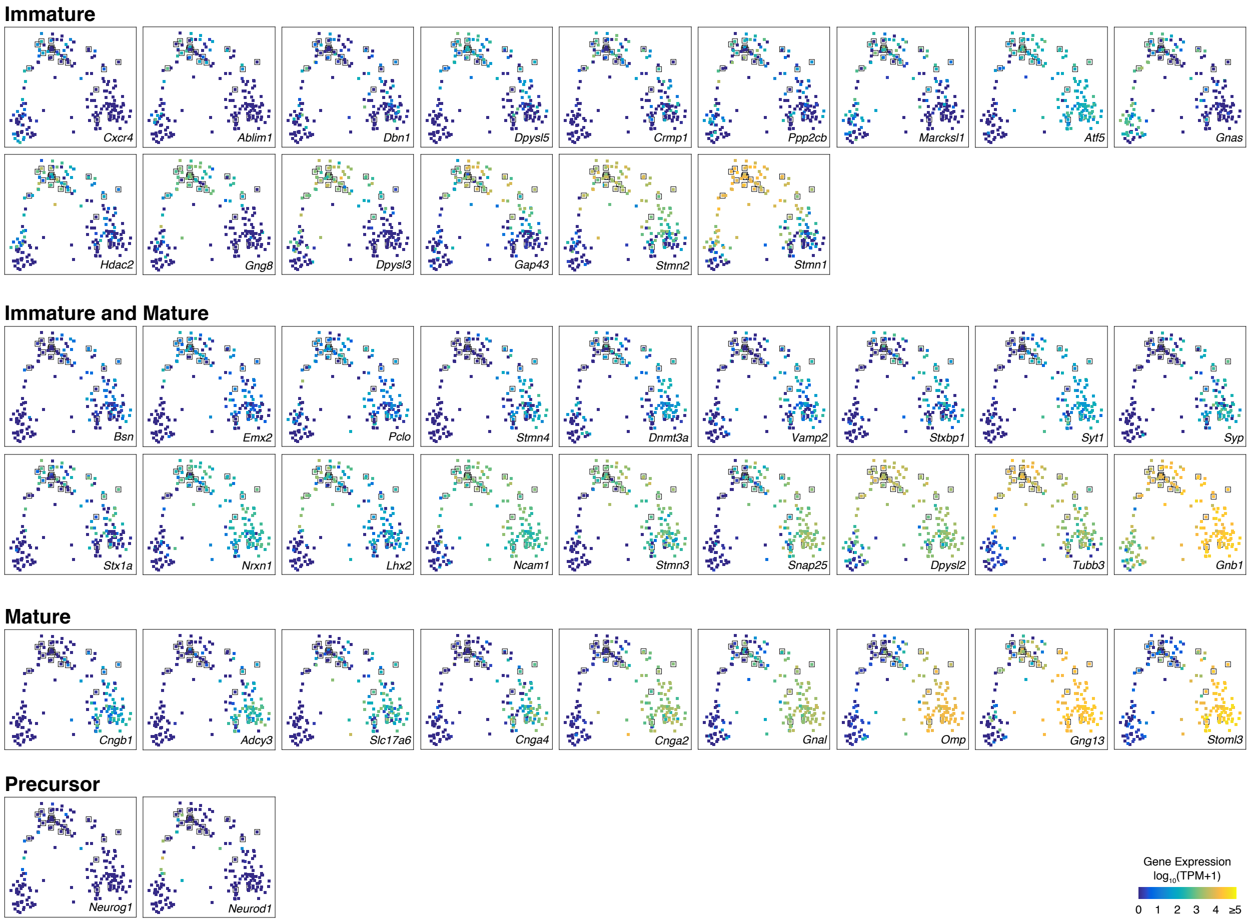

Appendix Figure S9. Expression profiles for 44 known marker genes (Fig. 1C) in all single cells. Horizontal and vertical axes are principle components 1 and 2, respectively. Genes are categorized and sorted in the same ways as in Fig. 1D. Multi-receptor neurons are marked by black boxes.
